# Supplementary material for: Elucidating the origin of chiroptical activity in chiral 2D perovskites through nano-confined growth
Source: Nat Commun. 2022 Jun 7;13:3259. doi: 10.1038/s41467-022-31017-9 (PMC9174244; doi:10.1038/s41467-022-31017-9)
Supplement: Supplementary file 1 — Supplementary Information [file 41467_2022_31017_MOESM1_ESM.pdf]

Supplementary Information for

Elucidating the origin of chiroptical activity in chiral 2D perovskites through nano-confined growth

Sunihl Ma[1], Young-Kwang Jung[1], Jihoon Ahn[1], Jihoon Kyhm[2], Jeiwan Tan,[1] Hyungsoo Lee,[1] Gyumin Jang,[1] Chan Uk Lee,[1] Aron Walsh[1,3], and Jooho Moon[1,]*

[1]Department of Materials Science and Engineering
Yonsei University
50 Yonsei-ro Seodaemun-gu, Seoul, 03722, Republic of Korea

[2]Technology Support Center
Korea Institute of Science and Technology
Seoul 02792, Republic of Korea

[3]Department of Materials
Imperial College London
London SW7 2AZ, UK

*Corresponding author: jmoon@yonsei.ac.kr

This PDF file includes:

Supplementary Note 1 to 4

Figure 1 to 16

References

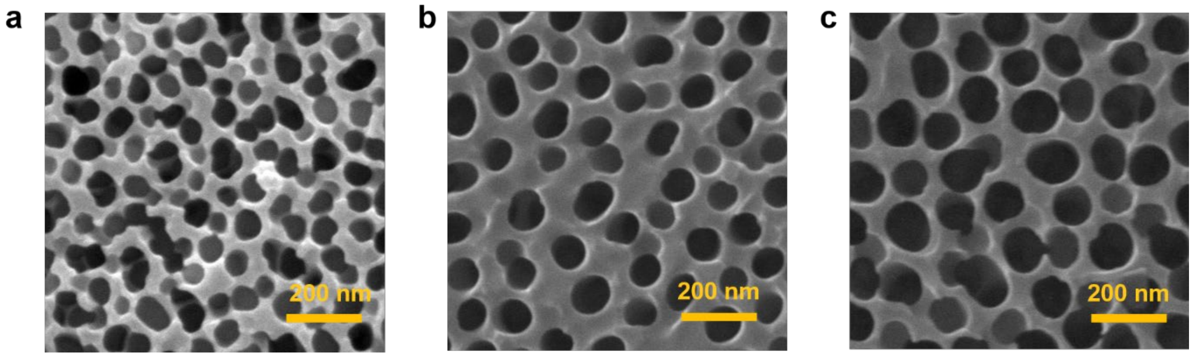

Supplementary Figure 1. Preparation of AAO templates with different pore sizes. Surface morphologies of AAO templates with a pore size of a, 66 nm, b, 100 nm, and c, 112 nm.

Fabrication of the AAO templates.

Fluorine-doped tin-oxide (FTO) coated glass substrates were washed with ethanol, deionized water, and acetone for 15 min and then exposed to UV treatment for 15 min. To form a TiO_2 blocking layer on FTO/glass substrate, the precursor solution (titanium isopropoxide (Sigma-Aldrich, 0.3408 mL) and a hydrochloric acid solution (0.042 mL) in ethanol (4 mL)) was spin coated on the FTO/glass substrate at 3000 rpm for 30 s, dried at 115 °C for 30 min, and then annealed at 500 °C in a box furnace for 1 h. Subsequently, the substrate was immersed in a 0.02 m diluted $TiCl_4$ (≥ 99%; Sigma-Aldrich) solution at 80 °C for 15 min and then annealed again at 500 °C for 30 min in a box furnace. After the substrate was cooled, Al was thermally evaporated on the TiO_2 blocking layer at a rate of 0.9 Å s^{-1} at a pressure below 8.3×10^{-6} Torr. The thickness of the deposited Al layers was controlled at 600 nm. The anodizing process was conducted in a diluted oxalic acid solution under constant DC 40 or 100 V at 6 °C. A widening process to expand the pore size of AAO was followed by immersing the sample in a diluted phosphoric acid solution at 30 °C. The pore size of the AAO was adjusted by varying the widening duration from 30 to 60 min.

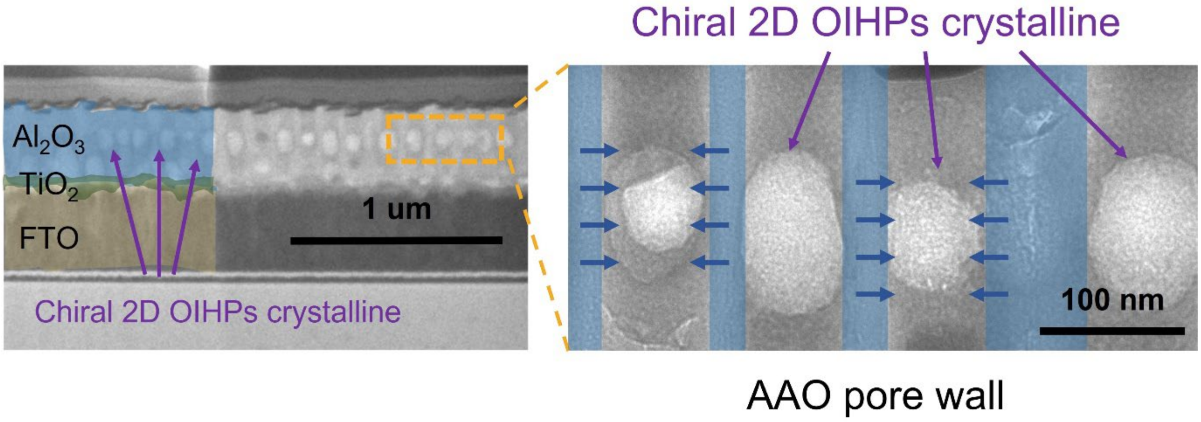

Supplementary Figure 2. The morphology of chiral 2D OIHPs grown inside AAO templates and high-resolution transmission electron microscopy (HRTEM) image of chiral 2D OIHPs confined in AAO templates with pore size of 66 nm condition.

As the lateral size of single crystal is limited by the pore size of AAO templates, we can calculate the average horizontal size of single crystal by analyzing the pore size distribution. By using ImageJ software (Wayne Rasband, National Institutes of Health, USA), we estimated the size distribution for three different AAO templates as 66.4 ± 1.3, 100.3 ± 3.9, and 112.7 ± 5.2 nm (Supplementary Figure 1). The image analysis confirms that the pore size of AAO templates (*i.e.*, lateral size of single crystalline chiral 2D OIHPs) exhibits a very narrow distribution.

Supplementary Note 1.

Reason and validity for choosing the halide composition.

Because the prevailing chirality transfer mechanism in chiral OIHPs is based on the inherent chiral crystal structure formation, it is imperative to elucidate the relationship between the crystal structure and chiroptic response to clarify the origin of chiroptical activity observed in chiral OIHPs. It was previously observed that the CD signal disappeared when the abrupt crystal structure transition occurred (*i.e.*, phase transition from the iodide-determinant phase (x = 0.3) to bromide-determinant phase (x = 0.4) in $MBA_2PbI_{4(1-x)}Br_{4x}$).[1] Although such a coincidence can provide a plausible explanation for interpreting the crystal structure-dependent chiroptical activity, in-depth crystallographic analysis is necessary to develop well-established chirality transfer mechanism in chiral OIHPs. Therefore, we began our investigation by focusing on the specific halide composition (x = 0.325) where the sudden phase transition from the iodide-determinant phase to bromide-determinant phase abruptly occurs. In addition, the composition range (from x = 0.325 to x = 0.400 with smaller interval of x = 0.025 than that of previous report[1]) where the distinct phase transition occurs was carefully scrutinized to precisely reveal the effects of crystal structure transition on the chiroptical activity of chiral 2D OIHPs.

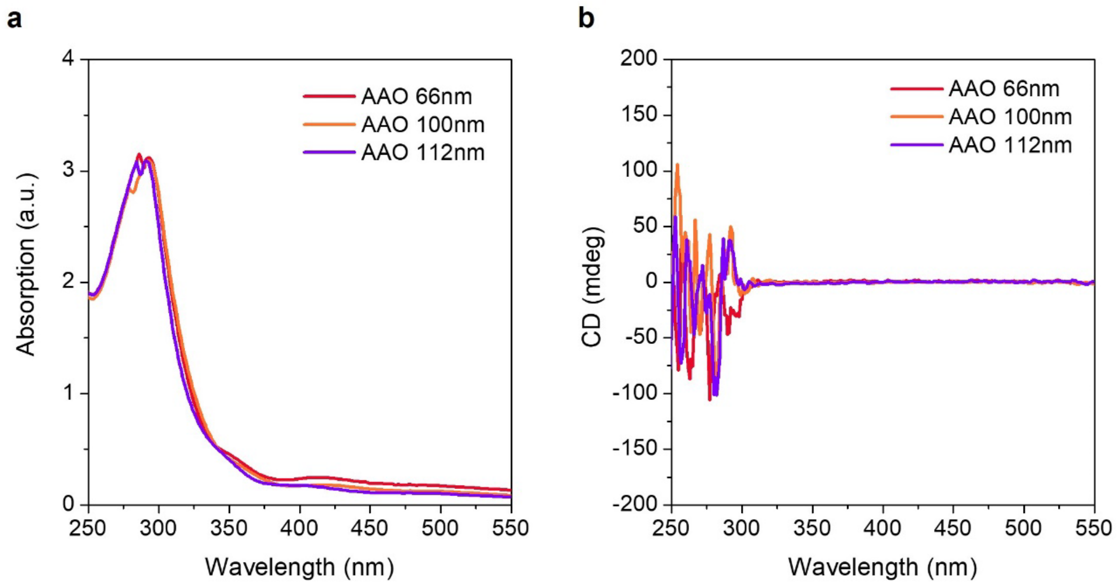

Supplementary Figure 3. Optical activity of bare AAO substrates with various pore size. **a**, Linear absorption spectra and **b**, CD spectra obtained from the empty AAO substrate without chiral 2D OIHPs.

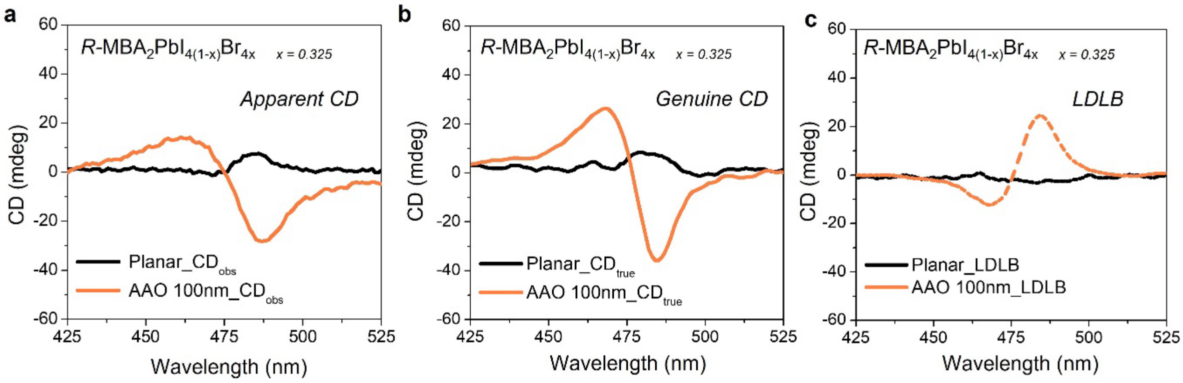

Supplementary Figure 4. The observed and calculated CD and LDLB spectra of chiral 2D perovskite grown on different substrate conditions. **a**, Apparent CD signal, **b**, genuine CD signal, and **c**, LDLB effect contribution calculated from the light direction dependent CD measurement.

Supplementary Note 2.

Validity and estimation of micro-strain in chiral OIHPs using the modified Williamson–Hall method.

Broadening and shifts of peaks in the XRD spectra can be induced by either a reduction in the grain size (Scherrer broadening) and/or non-uniform strain (micro-strain) and instrument-induced broadening. We note that Scherrer broadening would only be significant when the size of crystal is the main contribution of peak broadening and all other possible causes for micro-strain are negligible. In addition, as shown in Supplementary Figure 2, the vertical size of the crystal is larger than 100 nm. Therefore, we do not expect Scherrer broadening to be a significant contribution in chiral OIHPs. Micro-strain is the relative change in the size of materials with respect to its thermodynamic ideal size (or size before experiencing an external force). The micro-strain in a crystalline material is the result of small fluctuations in the lattice spacing induced by crystal imperfections, structural defects, including dislocations, vacancies, stacking faults, interstitials, twinning, and grain boundaries. In the case of chiral 2D OIHPs in AAO templates, the main source of peak broadening is micro-stress imposed by pore wall of AAO templates. By considering the Braggs law for the scattering of light of wavelength λ, $n\lambda = 2d\sin\theta$, it is clear that small fluctuations in *d*-spacing (*i.e.*, Δd) will result in small fluctuations or broadening in θ when measuring the XRD from the material. Consequently, we could quantify the degree of micro-strain in our chiral OIHPs thin films by analyzing the peak broadening in the diffraction patterns according to the modified Williamson–Hall method. The effective observed *d*-space broadening (Δd_{obs}) determined from the XRD peak width broadening is a convoluted function of the Gaussian full-width half-maximum broadening in the 2θ scan due to the instrument response ($\Delta d_{instrument}$; obtained from Supplementary Figure 5), the grain size (Δd_{size}), and the micro-strain (Δd_{ε}). These can be de-convoluted from the observed broadening via

$$\Delta d_{obs}^2 = \Delta d_{\varepsilon}^2 + \Delta d_{instrument}^2 + \Delta d_{size}^2 \quad (1)$$

where the unit-less micro-strain ε is defined as $\varepsilon = (\Delta d_{\varepsilon}/d)$, where d is the mean *d*-spacing. As mentioned above, the size effect-induced peak-width broadening can be neglected in chiral OIHPs thin films; hence, if $\Delta d_{size}^2 << \Delta d_{obs}^2$, we can write

$$(\Delta d_{obs}^2 - \Delta d_{ins}^2)^{1/2} \approx \varepsilon \cdot d \quad (2)$$

Therefore, the slope of the modified Williamson–Hall plot (*i.e.*, $(\Delta d_{obs}^2 - \Delta d_{instrument}^2)^{1/2}$ versus d) gives the magnitude of the micro-strain, ε, in the chiral OIHPs thin films.

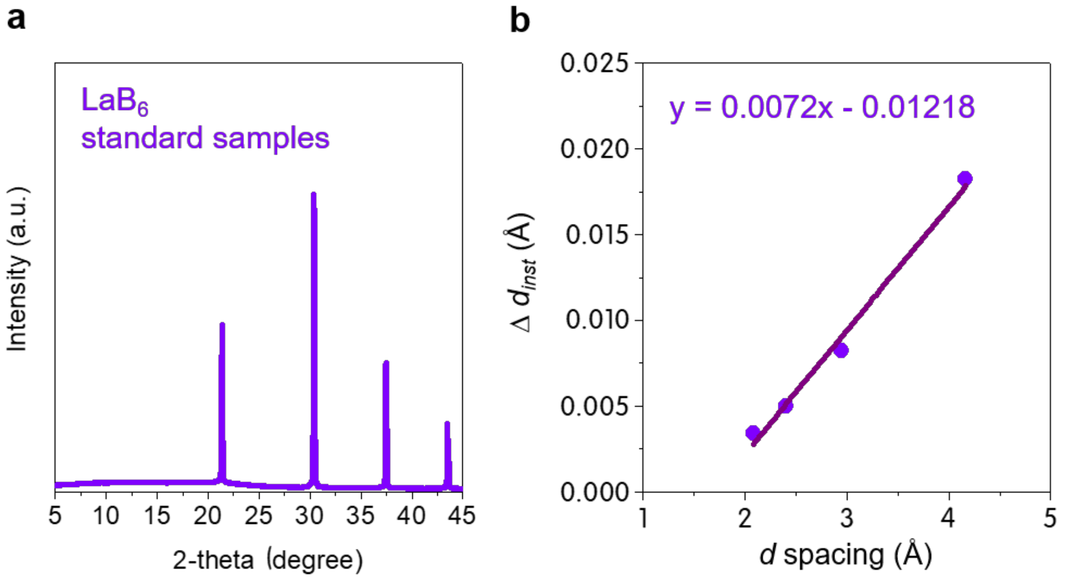

Supplementary Figure 5. Determination of micro-strain using modified Williamson–Hall method. **a**, powder XRD spectra of large-domain and low-strain LaB_6 standard sample for instrument peak-broadening calculation. **b**, A modified Williamson–Hall plot of $\Delta d_{instrument}$ as a function of d spacing extracted from the powder XRD spectra of the low-strain LaB_6 standard sample.

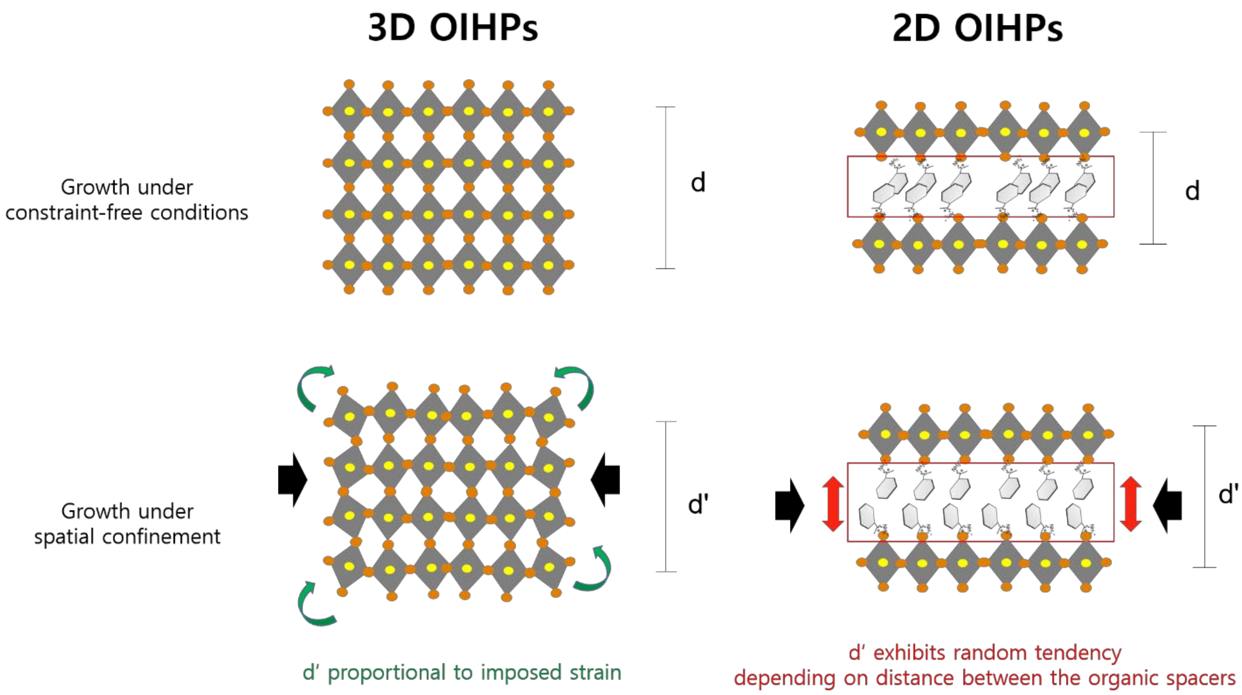

Supplementary Figure 6. Origin of the unprecedented dependency of micro-strain magnitude on pore size of AAO templates. Schematic illustration of different lattice shrinkage (or expansion) behaviors for 3D and 2D OIHPs under the spatially confined growth condition.

Supplementary Note 3.

Details and explanation for DFT calculation results for investigating the π–π stacking conformational change induced by micro-strain.

The calculated lattice constants of a_0 = 8.82 Å, b_0 = 9.18 Å, and c_0 = 28.63 Å are in good agreement with the experimental values of a_0^{exp} = 8.87 Å, b_0^{exp} = 9.25 Å, and c_0^{exp} = 28.73 in which measurement errors are within 1% (Supplementary Figure 7). It is worth to noting that a unit cell of *R*-MBA_2PbI_4 possess two distinguishable MBA^+ cations (MBA_1 and MBA_2) with delocalized π electrons (Supplementary Figure 8a). We postulate that two different directions of strain (*i.e.*, biaxial and uniaxial strain) can be imposed by AAO templates, depending on the relative angle between the crystal orientation of *R*-MBA_2PbI_4 and the pore wall. Based on the obtained micro-strain results, the DFT calculations were performed in a strain range from –6% (compressive) to +6% (tensile). As shown in Supplementary Figure 8b and c, regardless of the imposed strain directions, *R*-MBA_2PbI_4 exhibited drastic changes in lattice parameters, which induced the change in π–π stacking conformation (*e.g.*, angle and distance between the MBA_1 and MBA_2; see Supplementary Figure 8d and e). These calculations accentuate that the relative distance and angle between chiral organic spacers in chiral 2D OIHPs could be adjusted during the nanoconfined growth inside AAO templates.

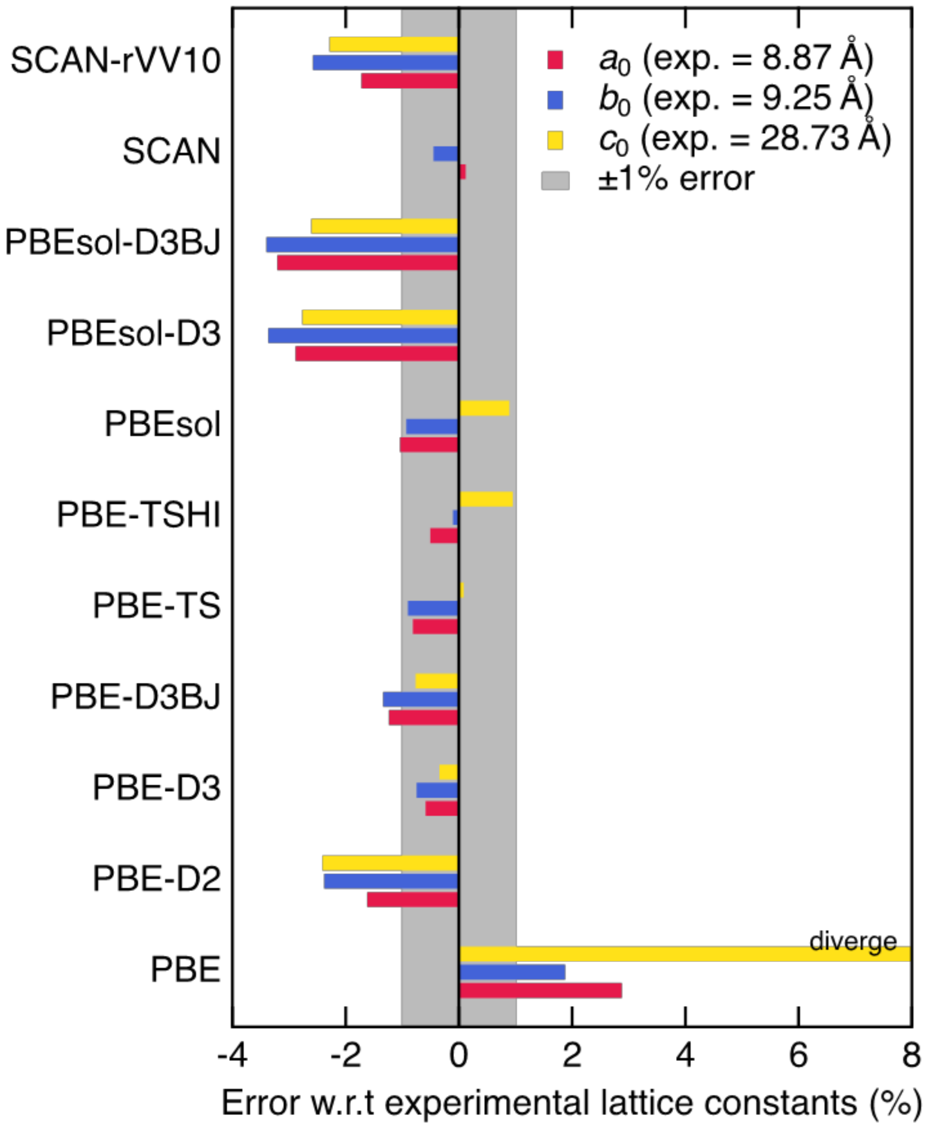

Supplementary Figure 7. Relative errors of calculated lattice constants from different exchange-correlation (xc) functionals and van der Waals (vdW) correction methods with respect to experimental values. PBE stands for Perdew–Burke–Ernzerhof exchange correlation functional,[2] PBEsol stands for PBE functional revised for solids,[3] and SCAN stands for strongly constrained and appropriately normed functional.[4] D2 denotes the DFT-D2 vdW correction method of Grimme,[5] D3 denotes the DFT-D3 method of Grimme's with zero damping,[6] D3BJ denotes the DFT-D3 method with Becke–Jonson damping,[7] TS denotes the Tkatchenko–Scheffler vdW correction method,[8] TSHI denotes the Tkatchenko–Scheffler method with iterative Hirshfeld partitioning,[9] and rVV10 denotes the revised Vydrov–van Voorhis nonlocal correlation functional.[10]

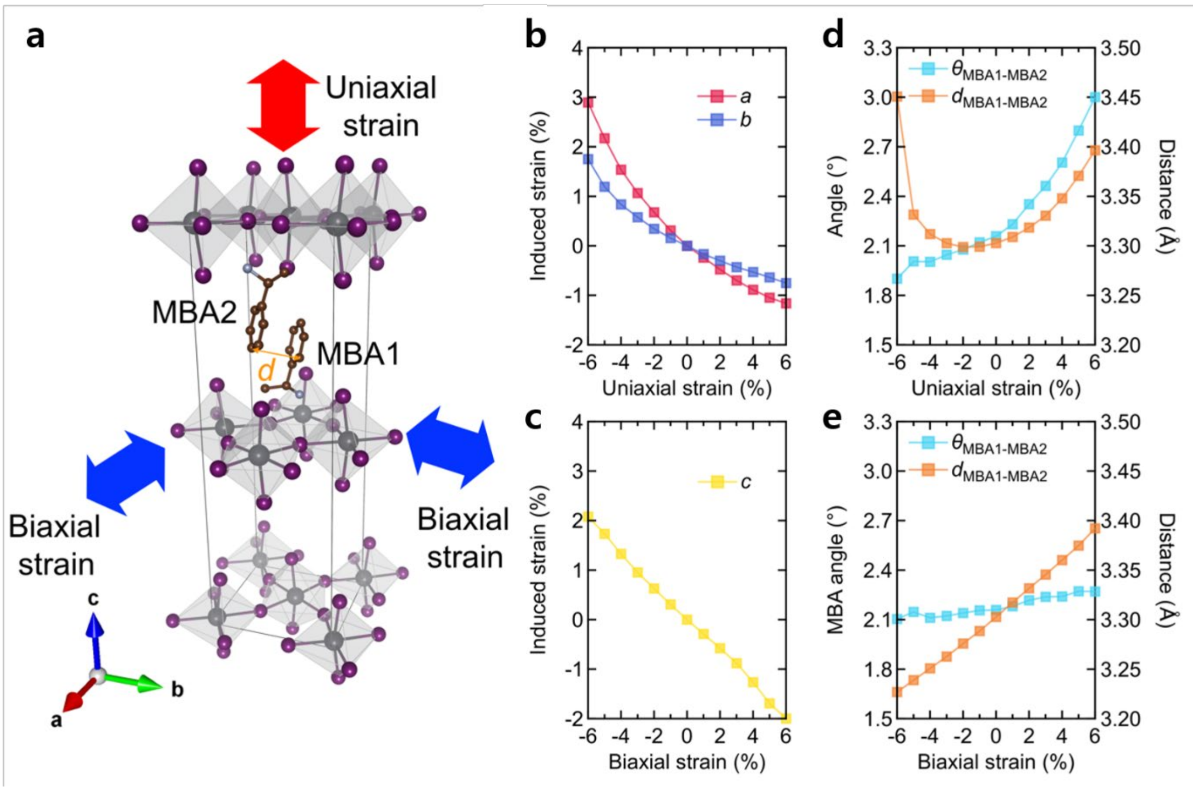

Supplementary Figure 8. DFT calculation results for investigating the π–π stacking conformation transition induced by micro-strain. a, Unit cell of R-MBA_2PbI_4. The red arrow indicates the direction of uniaxial strain applied to the unit cell, and the blue arrows represent the direction of biaxial strain applied to the unit cell. **b**, Induced strain along a- and b-axes under applied uniaxial strain. **c**, Induced strain along c-axis under applied biaxial strain. **d**, Changes in angle and distance between MBA_1 and MBA_2 in the unit cell under applied uniaxial strain. **e**, Changes in angle and distance between MBA_1 and MBA_2 in the unit cell under applied biaxial strain. The angle between MBA_1 and MBA_2 was obtained by measuring the angle between two planes that contain a benzene ring of MBA_1 and MBA_2, while the distance between MBA_1 and MBA_2 was obtained by measuring the shortest C–C distance between MBA_1 and MBA_2.

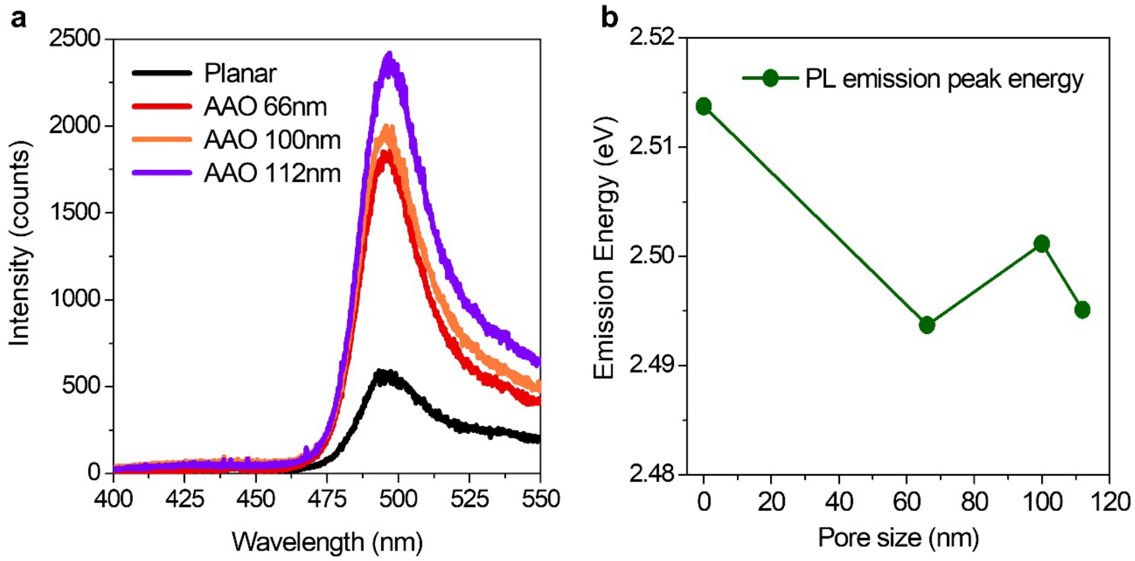

Supplementary Figure 9. The correlation between the AAO template pore sizes and induced micro-strain in chiral 2D OIHPs. **a**, Steady-state photoluminescence (PL) spectra of *R*-$MBA_2PbBr_xI_{4-x}$ (x=0.325) grown in various substrate conditions when excited by laser with wavelength of 325 nm. **b**, Corresponding the PL emission peak energy plot obtained from the steady-state PL spectra as a function of AAO template pore sizes.

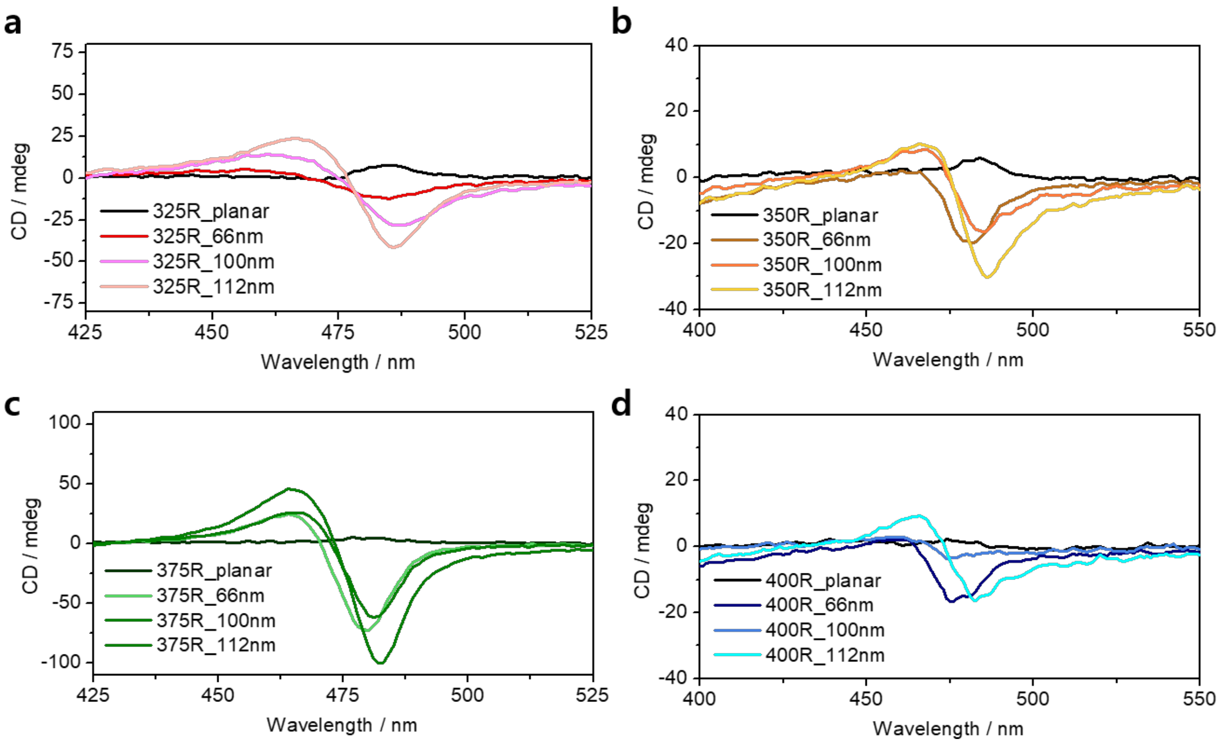

Supplementary Figure 10. Chiroptical activity of OIHPs grown on different substrate conditions with different halide compositions. CD spectra of *R*-$MBA_2PbI_{4(1-x)}Br_{4x}$ grown on different substrates condition (*i.e.*, planar and AAO templates with 66 nm, 100 nm, and 112 nm pore sizes); a, x = 0.325, **b**, x = 0.350, **c**, x = 0.375, and **d**, x = 0.400.

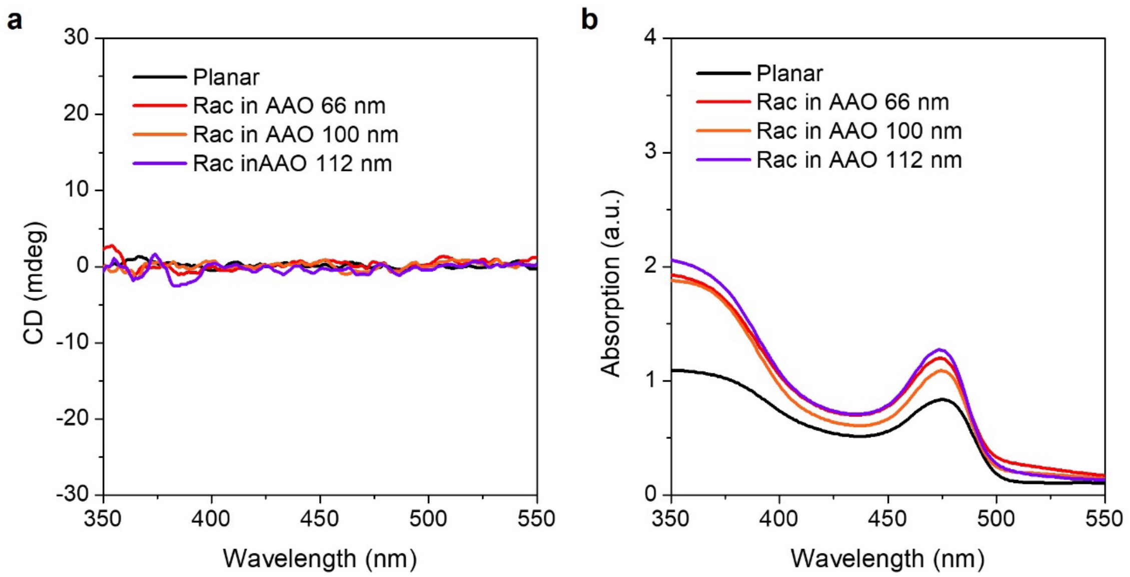

Supplementary Figure 11. Optical properties of racemic OIHPs grown in different substrate conditions. **a**, CD spectra and **b**, linear absorption spectra of racemic compound *R*-$MBA_2PbBr_xI_{4-x}$ (x=0.325) grown in different substrate conditions.

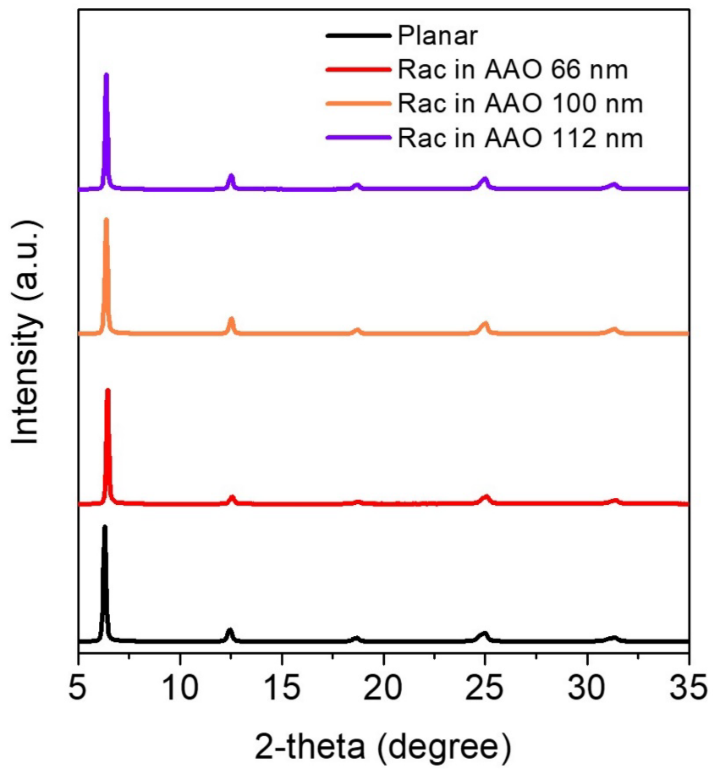

Supplementary Figure 12. XRD spectra of racemic compound *R*-$MBA_2PbBr_xI_{4-x}$ (x=0.325) grown in different substrate conditions.

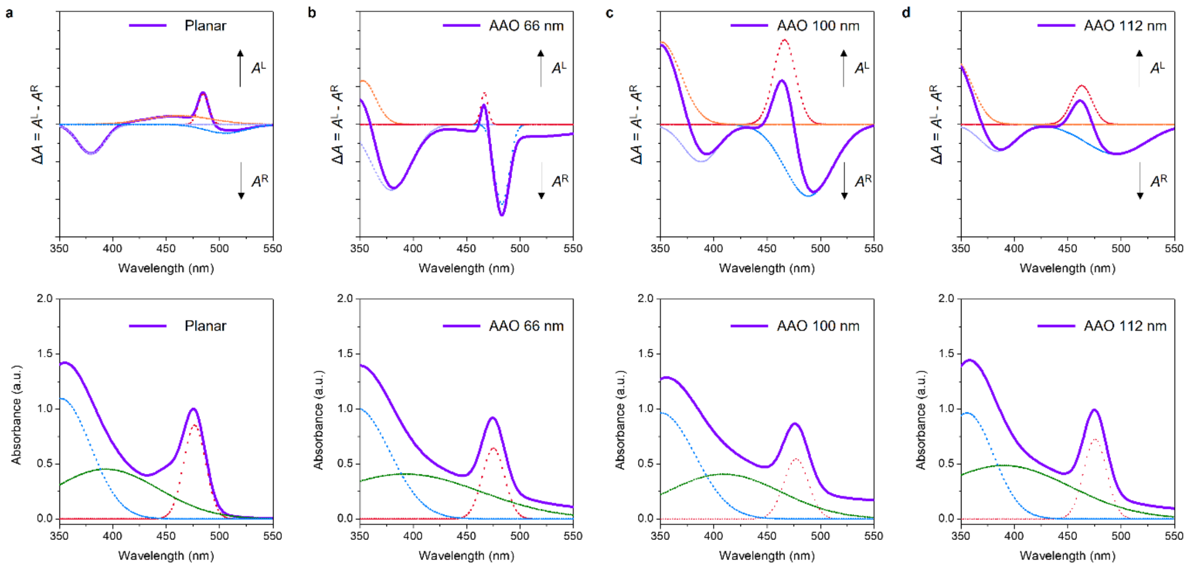

Supplementary Figure 13. Evaluation of the excited state splitting values from the deconvoluted CD spectra. Deconvolution results obtained from the CD spectra (upper panel) and corresponding extinction spectra (lower panel) of *R*-$MBA_2PbI_{4(1-x)}Br_{4x}$ chiral OIHPs (x = 0.325) grown on different substrate conditions; a, planar substrate, **b**, AAO template with a 66 nm pore size, **c**, AAO template with a 100 nm pore size, and **d**, AAO template with a 112 nm pore size. The solid purple line represents obtained CD spectra from the chiral 2D OIHPs. The red and blue dot-line indicate the absorption of LCP and RCP, respectively.

Supplementary Note 4.
Details on the fitting procedure

At first, the extinction spectrum of chiral OIHPs thin films was fitted by a sum of Gaussians using the least square means with fitting parameters of (A_{0i}, λ_{0i}, σ_i). The initial guess for the center was based on the minimums of the second derivatives of the absorption spectrum as an initial guess with tight borders. The initial values for amplitudes in the fit were estimated from the extinction at the center, and the width was approximated from the second derivative maxima that borders between each minimum. All absorption fits had high coefficients of determination: $R^2 > 0.997$.

Then, the CD spectrum was fitted by a linear combination of the derivatives of the Gaussians found by the extinction spectra decomposition. Although the chiral OIHPs exhibit multiple excitonic transition behaviors, the intensive excitonic transition behavior occurs near the first extinction band edge. Therefore, CD spectrum fitting was mainly conducted near the first extinction band edge. The dotted and bold lines (in Supplementary Figure 13) indicate the preferential absorption peak of LCP or RCP corresponding to each excitonic transition (Gaussians) and measured CD spectra, respectively. This result is demonstrated for only one halide composition (R-$MBA_2PbI_{4(1-x)}Br_{4x}$; x = 0.325) but works similarly for all other samples (*i.e.*, independent of grown substrates conditions and halide composition)

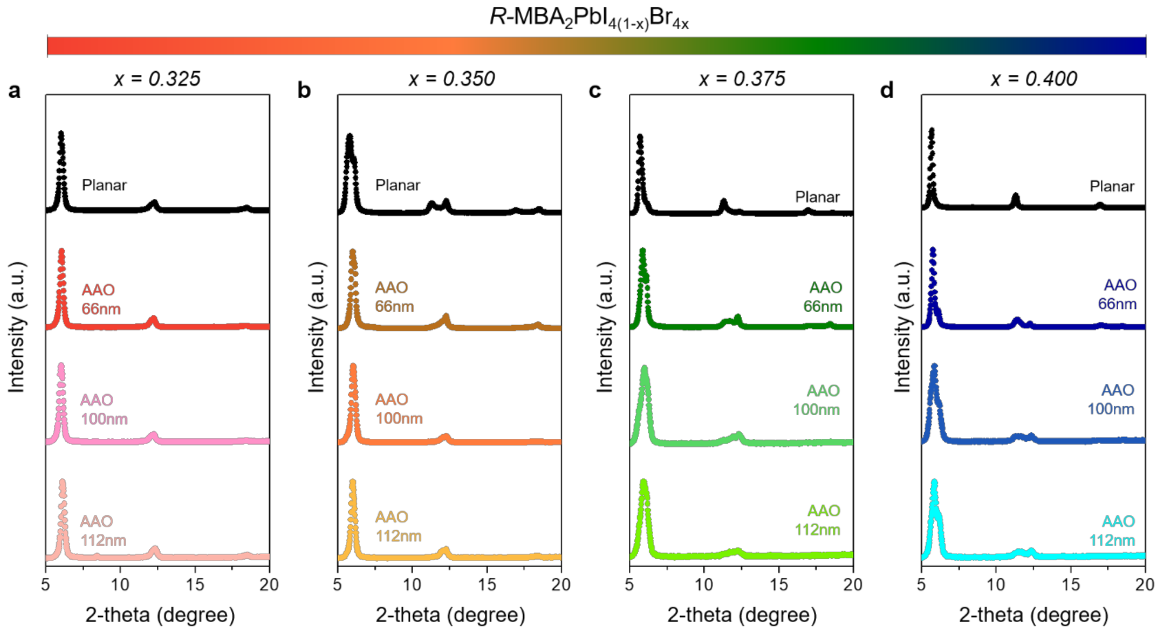

Supplementary Figure 14. Preferential orientation of chiral 2D OIHPs regardless of growing substrates and halide composition. Thin-film XRD patterns of chiral 2D OIHPs grown on different substrate conditions with different bromide compositions, **a**, x = 0.325, **b**, x = 0.350, **c**, x = 0.375, and **d**, x = 0.400.

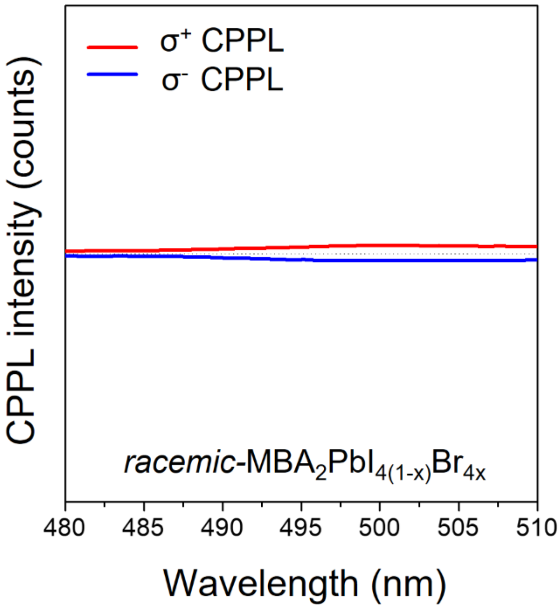

Supplementary Figure 15. CPPL spectra of racemic-$MBA_2PbI_{4(1-x)}Br_{4x}$ ($x = 0.325$) grown in AAO template with 100 nm pore size.

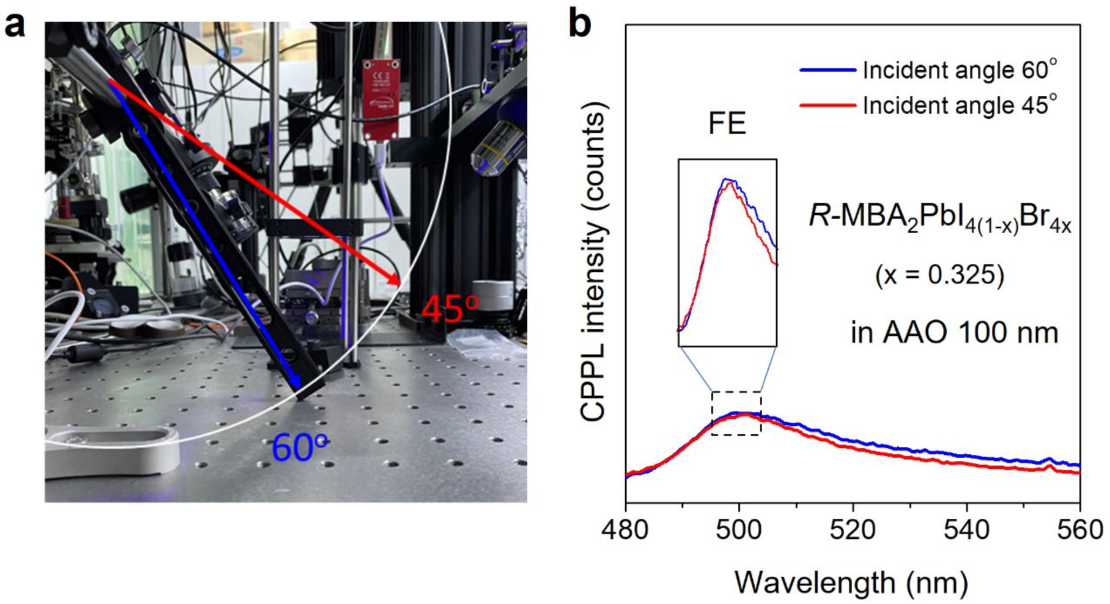

Supplementary Figure 16. a. A photograph of CPPL experimental setup with varying the incident angle of excitation laser. **b**. The CPPL spectra of R-$MBA_2PbI_{4(1-x)}Br_{4x}$ (x = 0.325) depending on the incident angle of excitation laser.

Supplementary References

1. Ahn, J. et al. Chiral 2D organic inorganic hybrid perovskite with circular dichroism tunable over wide wavelength range. *J. Am. Chem. Soc.* **142**, 4206–4212 (2020).

2. Perdew, J. P., Burke, K. & Ernzerhof, M. Generalized gradient approximation made simple. *Phys. Rev. Lett.* **78**, 1396–1396 (1997).

3. Perdew, J. P. et al. Restoring the density-gradient expansion for exchange in solids and surfaces. *Phys. Rev. Lett.* **102**, 039902 (2009).

4. Sun, J. W., Ruzsinszky, A. & Perdew, J. P. Strongly constrained and appropriately normed semilocal density functional. *Phys. Rev. Lett.* **115**, 036402 (2015).

5. Grimme, S. Semiempirical GGA-type density functional constructed with a long-range dispersion correction. *J. Comput. Chem.* **27**, 1787–1799 (2006).

6. Grimme, S., Antony, J., Ehrlich, S. & Krieg, H. A consistent and accurate ab initio parametrization of density functional dispersion correction (DFT-D) for the 94 elements H-Pu. *J. Chem. Phys.* **132**, 154104 (2010).

7. Grimme, S., Ehrlich, S. & Goerigk, L. Effect of the damping function in dispersion corrected density functional theory. *J. Comput. Chem.* **32**, 1456–1465 (2011).

8. Tkatchenko, A. & Scheffler, M. Accurate molecular van der Waals interactions from ground-state electron density and free-atom reference data. *Phys. Rev. Lett.* **102**, 073005 (2009).

9. Bucko, T., Lebegue, S., Hafner, J. & Angyan, J. G. Tkatchenko-Scheffler van der Waals correction method with and without self-consistent screening applied to solids. *Phys. Rev. B* **87**, 064110 (2013).

10. Sabatini, R., Gorni, T. & de Gironcoli, S. Nonlocal van der Waals density functional made simple and efficient. *Phys. Rev. B* **87**, 041108 (2013).
